# Use of Apps to Promote Childhood Vaccination: Protocol for a Systematic Review

**DOI:** 10.2196/16929

**Published:** 2020-02-05

**Authors:** Michelle Helena Van Velthoven, Madison Milne-Ives, Caroline de Cock, Mary Mooney, Edward Meinert

**Affiliations:** 1 Digitally Enabled PrevenTative Health (DEPTH) Research Group Department of Paediatrics Oxford United Kingdom; 2 School of Nursing & Midwifery Trinity College Dublin Dublin Ireland; 3 Department of Primary Care and Public Health Imperial College London London United Kingdom

**Keywords:** app, smartphone technology, vaccination, vaccines, immunization, children, mobile phone

## Abstract

**Background:**

The decline in the uptake of routine childhood vaccinations has resulted in outbreaks of vaccine-preventable diseases. Vaccination apps can be used as a tool to promote immunization through the provision of reminders, dissemination of information, peer support, and feedback.

**Objective:**

The aim of this review is to systematically review the evidence on the use of apps to support childhood vaccination uptake, information storage, and record sharing.

**Methods:**

We will identify relevant papers by searching the following electronic databases: PubMed, Embase by Ovid, Web of Science, Cochrane Central Register of Controlled Trials (CENTRAL), ClinicalTrials.gov, and Education Resources Information Center (ERIC). We will review the reference lists of those studies that we include to identify relevant additional papers not initially identified using our search strategy. In addition to the use of electronic databases, we will search for grey literature on the topic. The search strategy will include only terms relating to or describing the intervention, which is app use. As almost all titles and abstracts are in English, 100% of these will be reviewed, but retrieval will be confined to papers written in the English language. We will record the search outcome on a specifically designed record sheet. Two reviewers will select observational and intervention studies, appraise the quality of the studies, and extract the relevant data. All studies will involve the use of apps relating to child vaccinations. The primary outcome is the uptake of vaccinations. Secondary outcomes are as follows: (1) use of app for sharing of information and providing vaccination reminders and (2) use of app for storage of vaccination information; knowledge and decision making by parents regarding vaccination (ie, risks and benefits of vaccination); costs and cost-effectiveness of vaccination apps; use of the app and measures of usability (eg, usefulness, acceptability, and experiences of different users: parents and health care professionals); use of technical standards for development of the app; and adverse events (eg, data leaks and misinformation). We will exclude studies that do not study an app. We anticipate a limited scope for meta-analysis and will provide a narrative overview of findings and tabular summaries of extracted data.

**Results:**

This project was funded by the Sir David Cooksey Fellowship in Healthcare Translation at the University of Oxford, Oxford, United Kingdom. We will submit the full systematic review for publication in the *Journal of Medical Internet Research*.

**Conclusions:**

This review will follow, where possible, the Cochrane Collaboration and the Centre for Review and Dissemination methodologies for conducting systematic reviews. We will report our findings based on guidelines from the Preferred Reporting Items for Systematic Reviews and Meta-Analyses (PRISMA) statement. The review results will be used to inform the development of a vaccination app.

**International Registered Report Identifier (IRRID):**

PRR1-10.2196/16929

## Introduction

### Description of the Issue

Outbreaks of vaccine-preventable diseases, such as the measles, mumps, rubella, and diphtheria, have risen over the past decade [1-3]. While mortality rates of vaccine-preventable diseases are relatively low, certain groups, including children under 5 years of age and people with a compromised immune system, are at greater risk of severe complications [2]. The decline in the uptake of routine childhood vaccinations has been identified as a cause for outbreaks of vaccine-preventable diseases. Immunization coverage has declined for nine routine childhood vaccinations measured at different child ages in England; vaccination rates fell by 0.2%-1% in 2018-2019 compared to the previous year [4].

There are numerous interrelated reasons for the decline in childhood vaccinations, including concerns about side effects, fear of autism, objection against many injections, moral or religious grounds, costs, access, and other reasons [3]. A commonly mentioned reason is misinformation and false evidence, for example, claims by the discredited ex-physician Andrew Wakefield who linked the measles, mumps, and rubella (MMR) vaccine to autism in 1998 [5]. Religious and philosophical reasons have been used by certain groups to decline vaccination of their children [6]. Particular communities have been consistently difficult to engage, for example, male-dominated societies often resist vaccination against human papillomavirus (HPV) [7,8]. The seriousness and relative rarity of these illnesses has reduced some people’s awareness of the importance of vaccination. Visiting a health clinic for vaccinations might be an inconvenience or may also be forgotten about [3], particularly as reminders to attend a clinic for vaccinations are not part of routine health care in all countries.

### Description of the Intervention

There have been several initiatives to improve the uptake of childhood vaccinations in different settings [9,10]. These include a range of informational, behavioral, and environmental initiatives. Health care provider initiatives have focused on patient counseling, maximizing the opportunities of each visit, combination vaccines, and automated electronic patient record reminders. Community-based approaches to increase vaccination rates include increasing outreach and educational programs, using recall and reminder strategies, providing financial incentives, and offering vaccinations at nontraditional sites [3].

Over the past decade, public and private organizations have developed tools to improve vaccination coverage, including vaccination information websites and apps [11]. These apps help health care providers and patients to access reminders for recommended immunization schedules and related vaccine resources and websites; they also allow for changes in the schedules through app updates.

### How the Intervention Might Work

Vaccination apps can be used as a tool to provide reminders, information, peer support, and feedback [12]. A cluster randomized controlled trial showed that an app used by village doctors, which included text messages to caregivers, improved full vaccination coverage in China. Village doctors using the app reported improved efficiency in managing childhood vaccinations [13]. A quasi-experimental pre-post study using an app to electronically register child births and sending text message reminders to parents about upcoming vaccinations showed improved vaccination coverage in rural hard-to-reach and urban street dweller communities in Bangladesh [14].

### Why It Is Important to Do This Review

A systematic review that assessed interventions to improve immunization coverage in England concluded that current practice is insufficient [15]. Vaccination apps might be used to help improve immunization coverage but, to our knowledge, no recent systematic reviews have assessed the evidence on childhood vaccination apps. A systematic review on the design of a vaccination reminder app identified two publications on mobile apps, but the search was limited and conducted in 2015 [12]. Furthermore, this review did not assess all important quality indicators, including whether the app was secure, usable, engaging, efficacious, and cost-effective [16].

### Objective

Our objective is to systematically review the evidence on childhood vaccination apps by assessing the following:

1. The uptake of vaccination.
2. Knowledge and decision making by parents: risks and benefits of vaccination.
3. Costs and cost-effectiveness.
4. Use of the app and measures of usability (eg, usefulness, acceptability, and experiences of different users: parents and health care professionals).
5. Use of technical standards for development of the app.
6. Adverse events (eg, data leaks and misinformation).

## Methods

### Overview

This is the protocol for a systematic review of the literature that will be reported, where possible, according to the Preferred Reporting Items for Systematic Reviews and Meta-Analyses for Protocols (PRISMA-P), as provided in Multimedia Appendix 1 [17]. Our review will follow, where possible, the Cochrane Collaboration [18] and the Centre for Review and Dissemination [19] methodologies for conducting systematic reviews.

### Criteria for Considering Studies

#### Types of Studies

We will include observational studies, such as cross-sectional surveys, cohort studies, qualitative studies (eg, interview studies and focus groups), and intervention studies, such as randomized controlled trials and nonrandomized studies (eg, nonrandomized controlled trials, before-and-after studies, and interrupted-time-series studies). We will only include studies reported in English and published after 2008, when the first smartphone was launched.

#### Types of Participants

We will include studies involving children up to 18 years of age, the children’s parents or guardians, and health care providers in any country. We will exclude studies focusing on vaccination of adults.

#### Types of Interventions

We will include any studies assessing apps designed to support childhood vaccination uptake, information storage, and record sharing (see Table 1). We will exclude studies that do not involve the use or study of an app for childhood vaccinations and that solely focus on other ways of delivering vaccination interventions, such as text messaging, telephone calls, or a website [20,21].

**Table 1:** Childhood immunization schedule: Ireland example.

Child’s age	Where vaccination is given	Vaccine
Birth	Hospital or clinic	Bacille Calmette-Guerin (BCG) vaccine: a vaccine to protect against tuberculosis disease
2 months	General practitioner	6 in 1: vaccines against diphtheria, tetanus, whooping cough (ie, pertussis), polio, *Haemophilus influenzae* type b (Hib), and hepatitis B provided in one single injection Vaccines against pneumococcal disease, meningococcal B, and rotavirus disease
4 months	General practitioner	6 in 1: vaccines against diphtheria, tetanus, whooping cough (ie, pertussis), polio, *Haemophilus influenzae* type b (Hib), and hepatitis B provided in one single injection Vaccines against meningococcal B and rotavirus disease
6 months	General practitioner	6 in 1: vaccines against diphtheria, tetanus, whooping cough (ie, pertussis), polio, *Haemophilus influenzae* type b (Hib), and hepatitis B provided in one single injection Vaccines against pneumococcal disease and meningococcal C
12 months	General practitioner	Measles, mumps, and rubella (MMR) vaccine Vaccine against meningococcal B
13 months	General practitioner	Vaccines against meningococcal C, *Haemophilus influenzae* type b (Hib), and pneumococcal disease
4-5 years	General practitioner or school	4 in 1: vaccines against diphtheria, tetanus, whooping cough (ie, pertussis), and polio Measles, mumps, and rubella (MMR) vaccine
11-14 years	School	Tetanus and low-dose diphtheria and pertussis (Tdap) booster Meningococcal C booster Human papillomavirus (HPV) vaccine (2 doses)

#### Types of Comparators

We will include any type of comparator interventions.

#### Types of Outcome Measures

The primary outcome of this review is the uptake of vaccination. Secondary outcomes are knowledge and decision making by parents (ie, risks and benefits of vaccination); costs and cost-effectiveness; use of the app and measures of usability (eg, usefulness, acceptability, and experiences of different users: parents and health care professionals); use of technical standards for development of the app; and adverse events (eg, data leaks and misinformation).

### Information Sources

Relevant articles will be identified by searching the following electronic databases: (1) PubMed, (2), Embase through Ovid, (3) Web of Science, (4) Cochrane Central Register of Controlled Trials (CENTRAL) [22], (5) ClinicalTrials.gov, and (6) Education Resources Information Center (ERIC).

### Search Strategy

A draft search strategy can be found in Multimedia Appendix 1. This will be tailored to the different databases, with the assistance of a medical research librarian. No study design filter will be used, as both quantitative and qualitative studies are to be included. We will use the titles, abstracts, and keywords of a set of articles that we know meet our inclusion criteria to define a search strategy that will return all these articles without an unmanageably large number of irrelevant articles.

### Data Management, Collection, and Analysis

#### Selection of Studies

Studies that meet the inclusion criteria will be included in the review. Two reviewers will screen titles and abstracts against the inclusion and exclusion criteria. Where duplicates or publications from the same study are identified, articles will be screened and the more recent publication or the one with the most detail will be selected for inclusion in the review. Two reviewers will assess full texts for eligibility; any disagreement will be resolved through discussion with a third reviewer. Study selection will be demonstrated using a Preferred Reporting Items for Systematic Reviews and Meta-Analyses (PRISMA) flowchart.

#### Data Extraction

We will pilot the data extraction form on a small number of studies to develop the final data extraction form. One reviewer will extract data from the included studies, which will be validated by a second reviewer. The data extraction form will be based on the minimum requirements as recommended by the Cochrane Handbook for Systematic Reviews of Interventions [18]. The data extraction form will be comprised of a Microsoft Excel form and will include the following about the studies: general information (eg, title, authors, and date); characteristics (eg, study design, aim, duration, and inclusion and exclusion criteria); risk of bias, depending on study design; samples (eg, description, geographic location, and setting); interventions; outcomes, as specified above; and results (eg, outcomes and times of assessment).

#### Assessment of Methodological Quality and Risk of Bias

Quality assessment will be undertaken by two reviewers. Any disagreements will be resolved by consensus and by including the opinion of a third reviewer. The methods specified in the Cochrane Collaboration’s tool for assessing risk of bias will be used. Three bias assessment categories will be used: low, high, and unclear risk, as specified in the Cochrane Handbook for Systematic Reviews of Interventions [18]; as specified in this handbook [18], an adapted version of these domains will also be used for nonrandomized studies. For other types of studies, we will use adapted versions of the following: Cochrane’s Risk Of Bias In Non-randomized Studies-of Interventions (ROBINS-I) tool [23], the Critical Appraisal Skills Programme (CASP) tool for qualitative studies [24], and the Appraisal tool for Cross-Sectional Studies (AXIS) [25].

#### Assessment of Heterogeneity

We anticipate a limited scope for meta-analysis due to differences in study populations, interventions, and outcomes. If a sufficient number of studies are found, we will explore heterogeneity through consideration of the study populations, methods, and interventions by visual inspection of results. Also, in statistical terms, we will assess the chi-square test for homogeneity and the I^2^ statistic. We will define statistically significant heterogeneity as *P*<.10. The I^2^ will be assessed with the following levels of inconsistency: I^2^ of 0%-25% represents a low level of inconsistency; I^2^ of 26%-50% represents a moderate level of inconsistency; and I^2^>50% represents a high level of inconsistency.

#### Data Synthesis

If a meta-analysis is not possible, we will provide a narrative overview of the findings and tabular summaries of extracted data. If a meta-analysis can be performed, this will allow us to estimate a summary measure of effect on relevant outcomes. For dichotomous outcomes, odds ratios will be used as the summary statistic. For continuous outcomes, mean difference will be the summary statistic. Standard pairwise meta-analysis will be conducted when more than one randomized controlled trial is identified.

#### Subgroup Analyses

If appropriate, we will provide a narrative overview of subgroups, including different interventions, participants, and geographic regions.

## Results

This project was funded by the Sir David Cooksey Fellowship in Healthcare Translation at the University of Oxford, Oxford, United Kingdom. We will submit the full systematic review for publication in the *Journal of Medical Internet Research*.

## Discussion

We will systematically review the evidence on apps to facilitate the vaccination process. Our review will follow, where possible, the Cochrane Collaboration and the Centre for Review and Dissemination methodologies for conducting systematic reviews. We will report our findings based on guidelines from the PRISMA statement. A comprehensive search of the evidence will be conducted. A potential limitation of this review is that the quality and quantity of studies using similar methods and interventions may be limited. The review results will be used to inform the development of a vaccination app.

## Appendix

Multimedia Appendix 1 Reporting checklist based on the Preferred Reporting Items for Systematic Reviews and Meta-Analyses for Protocols (PRISMA-P) guidelines and draft searches.

## Abbreviations

AXIS: Appraisal tool for Cross-Sectional Studies
CASP: Critical Appraisal Skills Programme
CENTRAL: Cochrane Central Register of Controlled Trials
ERIC: Education Resources Information Center
HPV: human papillomavirus
JMIR: *Journal of Medical Internet Research*
MMR: measles, mumps, and rubella
PRISMA: Preferred Reporting Items for Systematic Reviews and Meta-Analyses
PRISMA-P: Preferred Reporting Items for Systematic Reviews and Meta-Analyses for Protocols
ROBINS-I: Risk Of Bias In Non-randomized Studies-of Interventions
